# Relationship between cervical elastography and spontaneous onset of labor

**DOI:** 10.1038/s41598-020-76753-4

**Published:** 2020-11-12

**Authors:** Yoshie Yo, Yasushi Kotani, Reona Shiro, Kiko Yamamoto, Risa Fujishima, Hisamitsu Takaya, Ayako Suzuki, Masao Shimaoka, Noriomi Matsumura

**Affiliations:** Department of Obstetrics and Gynecology, Kindai University Faculty of Medicine, 377-2, Ohno-higashi, Osaka-sayama, Osaka, Japan

**Keywords:** Medical research, Signs and symptoms

## Abstract

Cervical elastography might be an objective method for evaluating cervical ripening during pregnancy, but its usefulness has not been fully investigated. We examined the significance of cervical elastography in the last trimester of pregnancy. Cervical elastography was performed at weekly checkups after 36 weeks of gestation in 238 cases delivered at our hospital from 2017 to 2018. The correlation with the onset time of natural labor, which is an index for judging maternal delivery preparation status, was examined. A total of 765 examinations were conducted, and cervical stiffness determined by cervical elastography was positively correlated with the Bishop score (r = 0.46, p < 0.0001). When examined separately for each week, only the examinations performed at 39 weeks were associated with the onset of spontaneous labor up to 7 days later (p = 0.0004). Furthermore, when stratified and analyzed by the Bishop score at 39 weeks of gestation, cervical elastography was associated with the occurrence of spontaneous labor pain for up to seven days in the groups with Bishop scores of 3–5 and 6–8 (p = 0.0007 and p = 0.03, respectively). In conclusion, cervical elastography at 39 weeks of pregnancy is useful for judging the delivery time.

## Introduction

Assessing maternal labor readiness is a basic matter of pregnancy and delivery management. Predicting the onset of spontaneous labor is important to encourage pregnant women to prepare for labor. The Bishop score^1^, as determined by pelvic examination, has been used to evaluate the preparatory state of delivery. However, this pelvic examination is subjective, and the error between examiners is large. Additionally, the pelvic examination fingers cannot reach in some cases, and pain and discomfort might occur, necessitating a more objective and less invasive evaluation method.

During pregnancy, in the mature cervical canal, neutrophil infiltration is observed, and local cytokines are produced. These processes cause the swollen cervical tissue to soften^2,3^. Cervix ripening results in ease of cervix opening and forms the birth canal. Moreover, cytokine production and decreased progesterone activity also cause uterine contractions and are associated with labor onset^4^.

Elastography is a technique that shows the hardness of the tissue in color and can be divided into strain elastography and shear wave elastography. Elastography has been useful for the diagnosis of breast cancer and liver cirrhosis and is used in daily practice. An elastographic evaluation of cervical softness using a transabdominal probe has also been reported^5–7^.

Furthermore, since 2007, cervical elastography has been performed using a transvaginal probe, and its clinical application is expected. Cervical elastography has been useful for predicting subsequent premature birth and vaginal delivery during the induction of labor. However, another report did not predict the success rate of transvaginal delivery^8^.

We consider that cervical elastography as an indicator of the preterm birth rate and success rate of labor induction is not a pure evaluation index because it is affected by the treatment, size of the bone production tract, fetal size, and fetal well-being, among others; it does not merely evaluate cervical ripening. This study examined the correlation between cervical elastography and pelvic examination findings in the third trimester. In addition, the significance of cervical elastography for predicting the onset of spontaneous labor was demonstrated.

## Results

### The relationship between the Bishop score and the number of days

**Figure 1:**
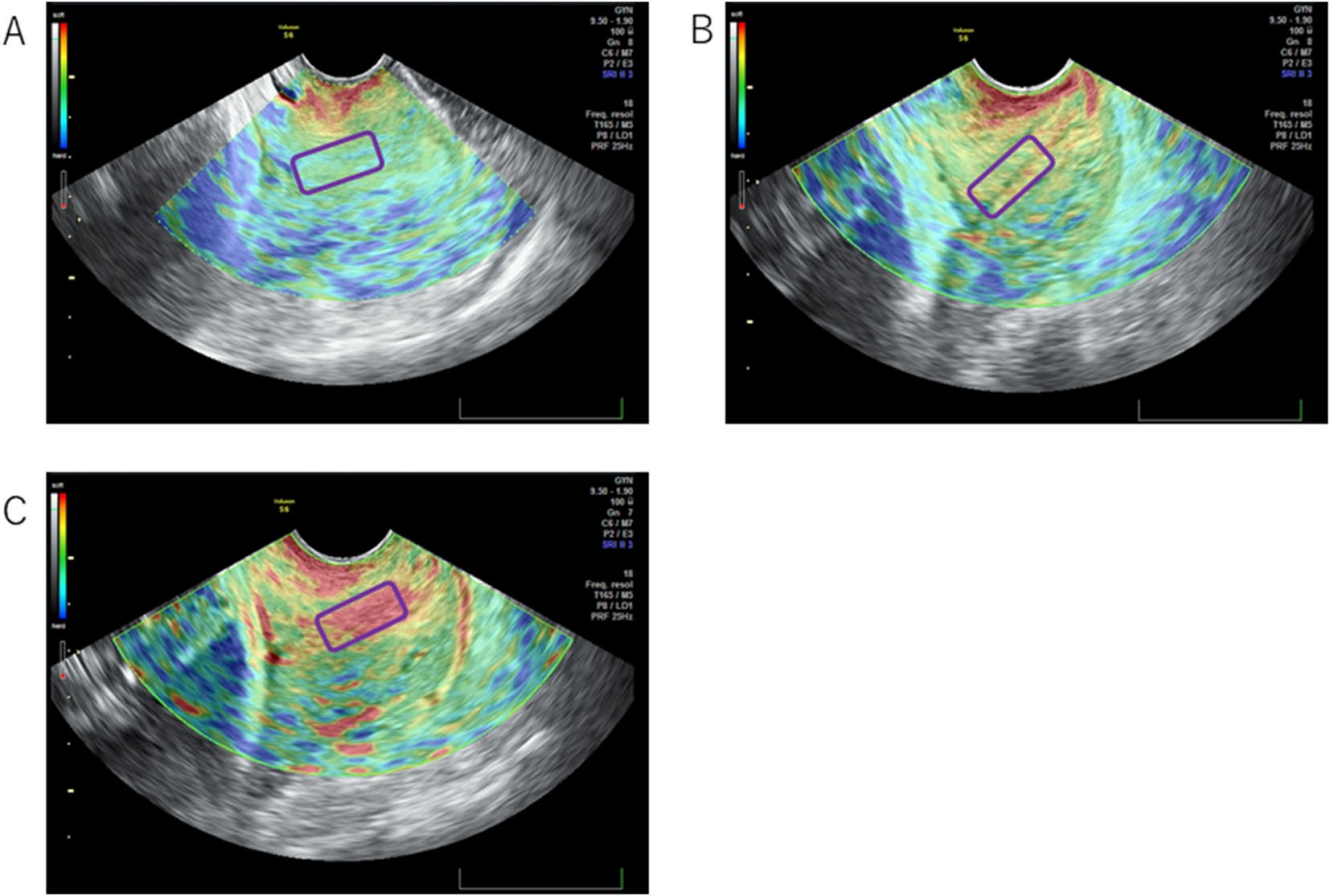
Representative images of cervical elastography (VOLUSONS6) in multiple pregnant women. The inner uterine ostium of the cervix (area surrounded by a purple square) was evaluated. (**A**) Green, hard tissue; (**B**) yellow, medium-hard tissue; (**C**) red, soft tissue.

First, we examined the relationship between the Bishop score and the number of days until the onset of spontaneous labor. The highest recorded Bishop score was 8 points. We divided the Bishop score into three groups: 0–2 points, 3–5 points, and 6–8 points. After examining the incidence of spontaneous labor within 7 days from the pelvic examination, we determined that those with a higher Bishop score tended to have a higher rate of spontaneous labor onset. There was a significant difference at 37 and 39 weeks of gestation (p = 0.01 and p = 0.02, respectively; Fig. 1). In addition, when examining the number of days from the pelvic examination to the onset of spontaneous labor, not limited to 7 days, higher Bishop scores tended to be associated with the onset of labor, particularly at the 39th week of pregnancy (p = 0.0007, Supplementary Figure S1).

### Elastography and scoring of pelvic examination findings

The correlation between cervical elastography and its internal findings was examined by Spearman’s correlation. In the total 769 tests, the correlations between the Bishop score, stiffness, dilatation, effacement, and elastography were strong (r = 0.46, 0.30, 0.39, and 0.46, respectively), and the correlations between position and station with elastography were weak (r = 0.08, 0.10, respectively) (Table 1).

**Table 1:** Correlation between cervical elastography findings and pelvic examination findings.

	Bishop score	Stiffness	Dilatation	Effacement	Position	Station
All	r = 0.46 p < 0.0001	r = 0.30 p < 0.0001	r = 0.39 p < 0.0001	r = 0.46 p < 0.0001	r = 0.08 p = 0.03	r = 0.10 p = 0.005
36 weeks	r = 0.42 p < 0.0001	r = 0.24 p = 0.001	r = 0.38 p < 0.0001	r = 0.41 p < 0.0001	r = 0.11 p = 0.12	r = 0.04 p = 0.55
37 weeks	r = 0.40 p < 0.0001	r = 0.27 p < 0.0001	r = 0.36 p < 0.0001	r = 0.48 p < 0.0001	r = − 0.003 p = 0.96	r = 0.03 p = 0.63
38 weeks	r = 0.47 p < 0.0001	r = 0.32 p < 0.0001	r = 0.32 p < 0.0001	r = 0.51 p < 0.0001	r = 0.06 p = 0.41	r = 0.08 p = 0.29
39 weeks	r = 0.36 p < 0.0001	r = 0.28 p = 0.002	r = 0.28 p = 0.002	r = 0.39 p < 0.0001	r = 0.08 p = 0.40	r = 0.10 p = 0.29
40 weeks	r = 0.35 p = 0.003	r = 0.34 p = 0.004	r = 0.33 p = 0.006	r = 0.34 p = 0.004	r = − 0.07 p = 0.58	r = 0.04 p = 0.77
Voluson	r = 0.44 p < 0.0001	r = 0.32 p < 0.0001	r = 0.35 p < 0.0001	r = 0.44 p < 0.0001	r = 0.09 p = 0.02	r = 0.07 p = 0.10
ARIETTA	r = 0.55 p < 0.0001	r = 0.33 p < 0.0001	r = 0.59 p < 0.0001	r = 0.55 p < 0.0001	r = 0.02 p = 0.82	r = 0.19 p = 0.02

To quantify the elastography findings, the categorical variables were defined as: 0 for green, 1 for yellow, and 2 for red. Stiffness was quantified as: 0 for hardness, 1 for moderate, and 2 for soft. The position of the cervix was quantified numerically: 0 for posterior, 1 for the middle, and 2 for anterior. The effacement (%), dilation (cm), station (cm), and Bishop score of the pelvic examination findings were used as their actual values. The correlation between elastography and the internal examination findings (during the same examination) were analyzed using a Spearman’s correlation.

When examined by week, the cervical elastography findings were positively correlated with the Bishop score, stiffness, dilatation, and effacement (r = 0.24 ~ 0.51), but there was only a weak correlation with position and station at any week (r = − 0.07 ~ 0.11) (Table 1).

When examining each ultrasonic model separately for VOLUSONS6 (January 2017 to May 2018) and ARIETTA 60 (June 2018 to December 2018), both showed that the Bishop score, stiffness, dilatation, and effacement were strongly correlated with elastography (all r > 0.30), but position and station were only weakly correlated with elastography (all r < 0.20) (Table 1).

### Comparison of the number of days from the examination date to the onset of natural labor

**Figure 2:**
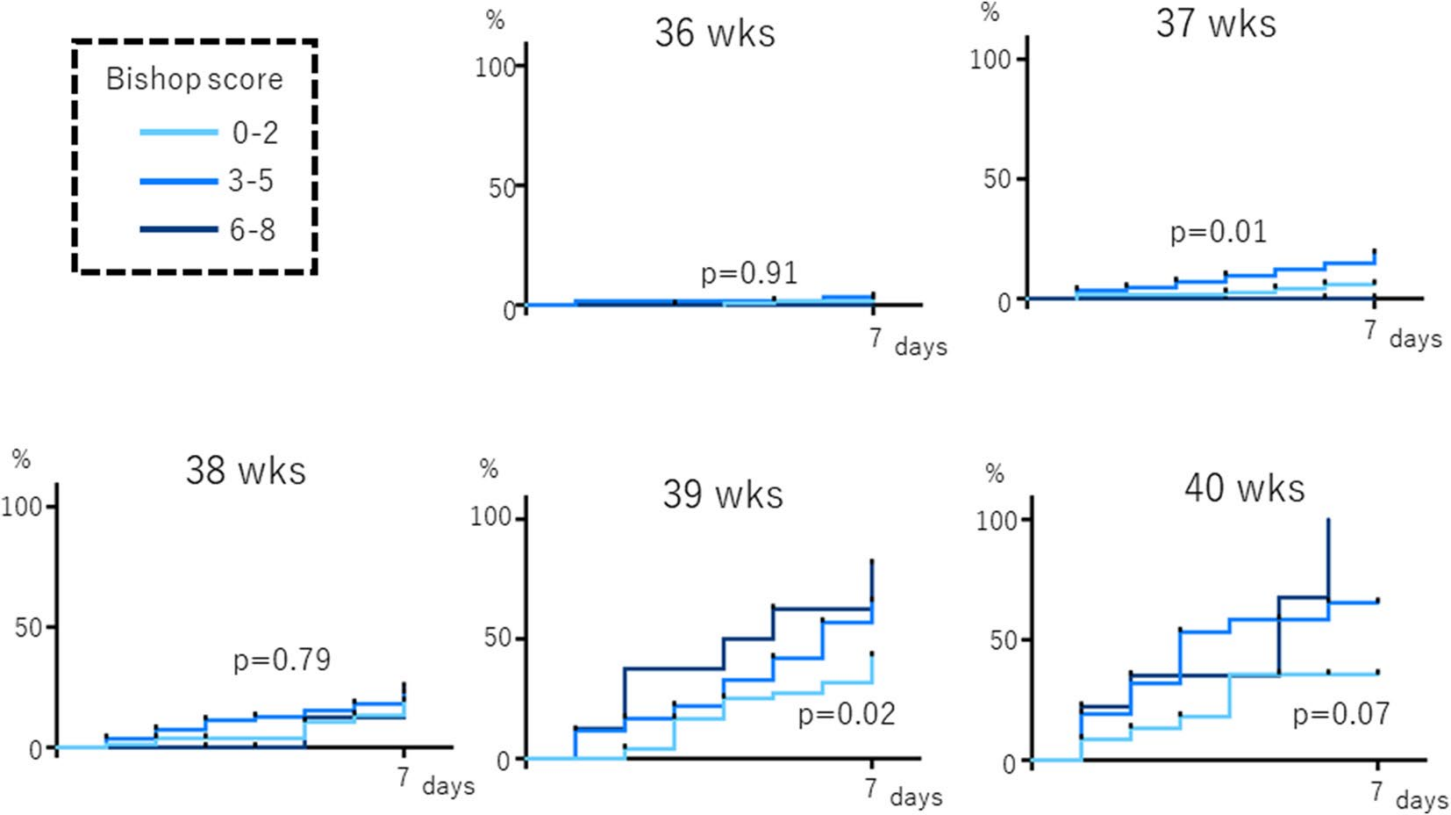
Association between the Bishop score and incidence of spontaneous labor onset within 7 days after the exam. Palatal axis; cumulative spontaneous labor onset rate. Horizontal axis: days since inspection; wks, weeks of gestation.

We investigated whether the Bishop score performed from 36 to 40 weeks of pregnancy (classified as scores of 0–2, 3–5, and 6–8) correlated with the onset of spontaneous labor within 7 days after the exam. Only at week 39, there was a significant difference between the three groups between the Bishop scores (p = 0.02, Fig. 2).

We also investigated which cervical elastography out of those performed from 36 to 40 weeks of pregnancy was correlated with the onset of spontaneous labor.

**Figure 3:**
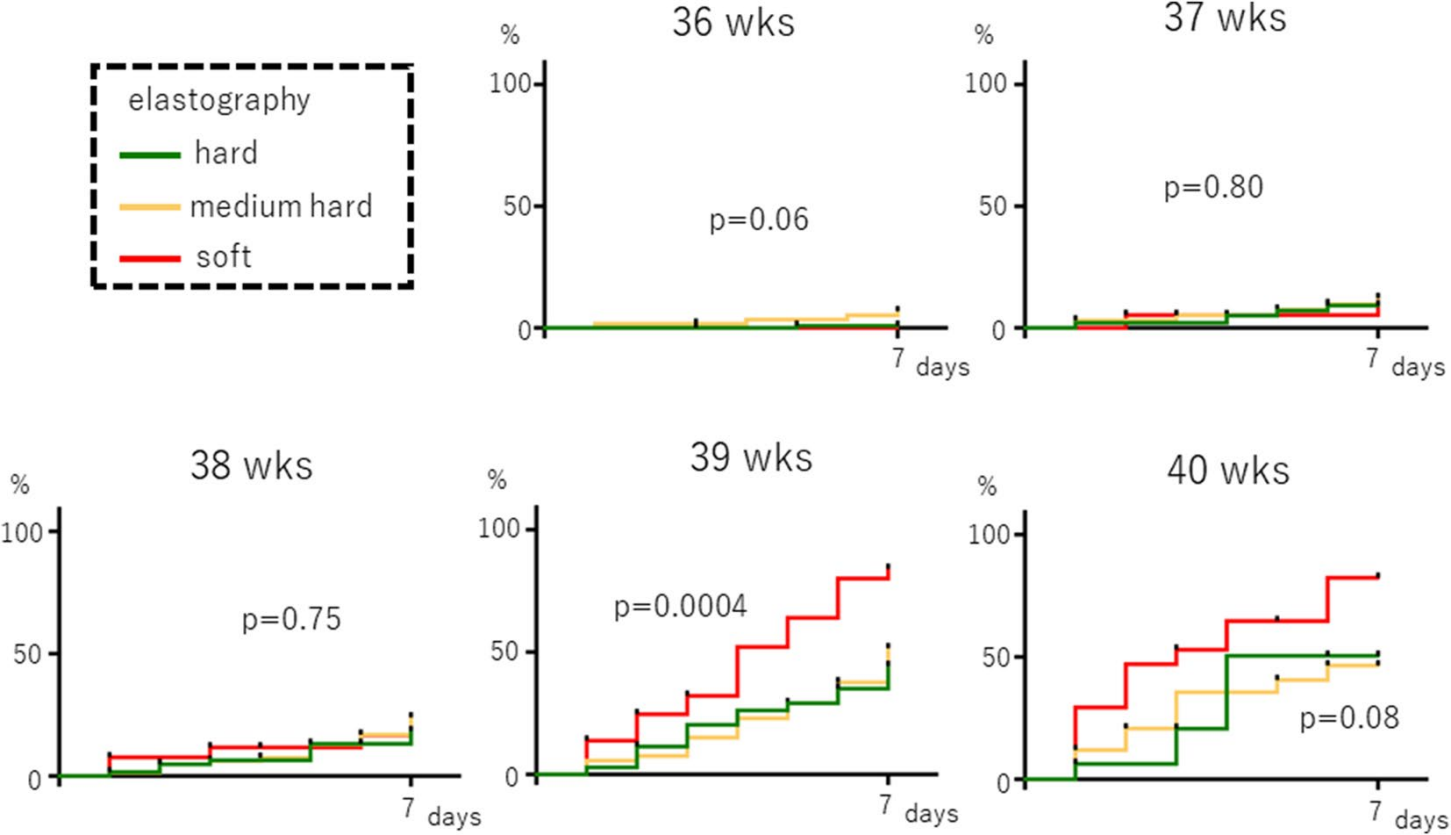
Association between cervical elastography and the incidence of spontaneous labor within 7 days after the exam. Palatal axis; cumulative spontaneous labor onset rate. Horizontal axis: days since the inspection.

The relationship between cervical elastography and the incidence of spontaneous labor within 7 days of the examination showed that labor tended to occur earlier in the soft tissue group at 39 weeks of gestation, and a significant difference was noted among the three groups: the rate of labor onset was 80% in the soft tissue group and 40% in the medium-hard tissue and hard tissue groups at seven days after 39 gestational weeks (p = 0.0004, Fig. 3). At 40 weeks, the rate of spontaneous labor onset within 7 days also tended to be high, but there was no significant difference (p = 0.08, Fig. 3). Similarly, when examining the number of days from the exams to spontaneous labor onset, not limited to 7 days, labor onset occurred significantly earlier in the soft tissue group than in the medium-hard tissue or hard tissue groups, but this finding was only observed at 39 weeks gestation (p = 0.001, Supplementary Figure S2).

**Figure 4:**
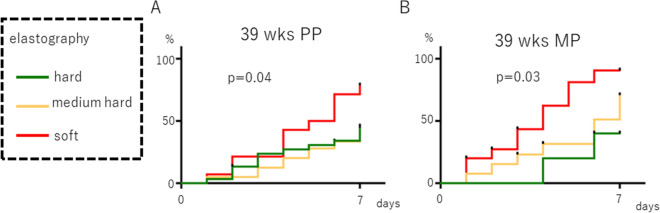
Association between cervical elastography at 39 weeks of gestation and the incidence of spontaneous labor within 7 days after the exam. (**A**) primipara (PP), (**B**) multipara (MP). Palatal axis; cumulative spontaneous labor onset rate. Horizontal axis: days since the inspection.

When analyzing the incidence of spontaneous labor onset within 7 days of the examination at the 39th week of pregnancy, divided into primiparas and multiparas, labor onset appeared early when the elastography findings were classified as soft tissue, regardless of primiparas and multiparas. A statistically significant difference was observed (p = 0.04 and p = 0.03, respectively, Fig. 4).

**Figure 5:**
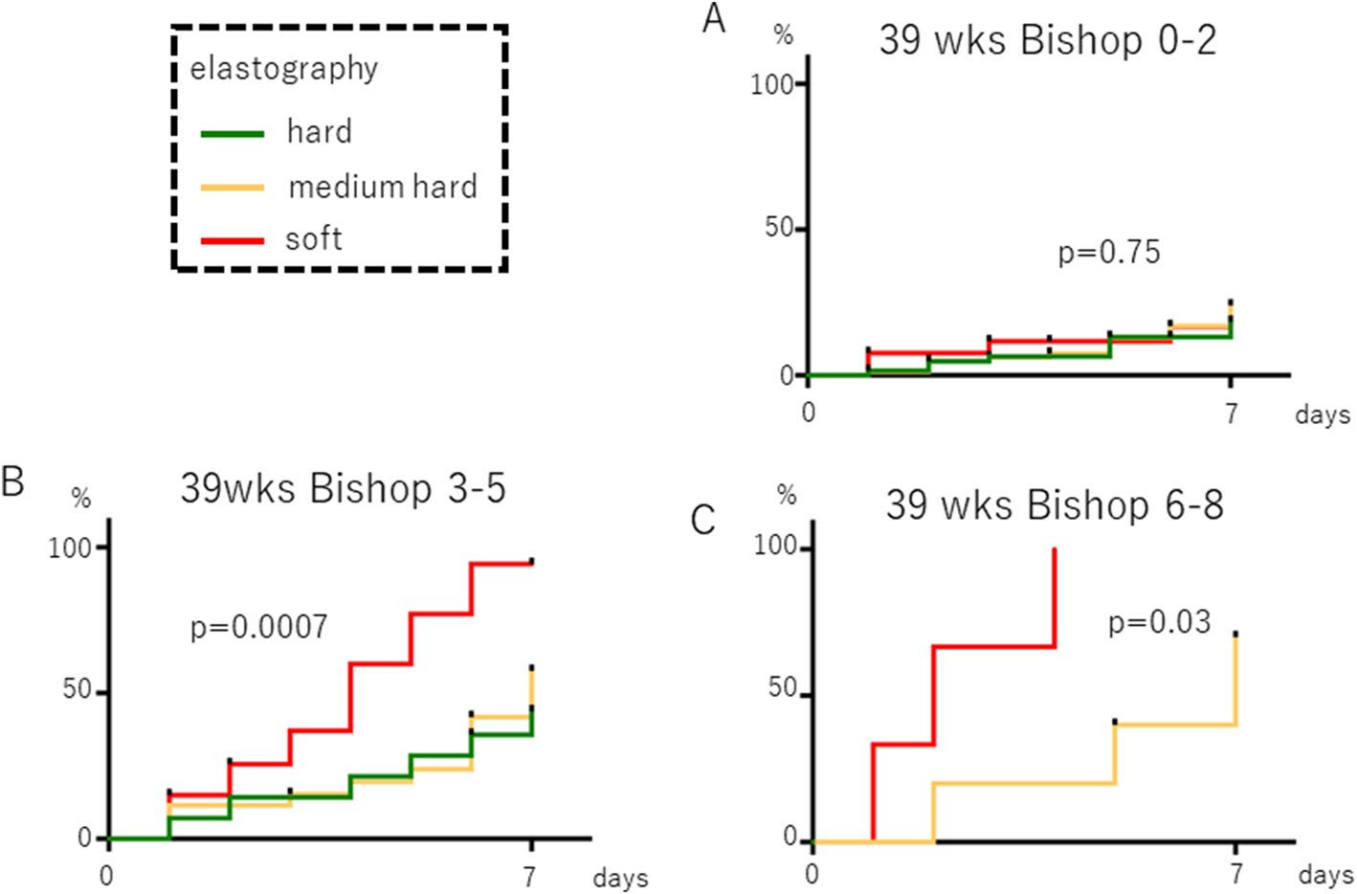
Association between cervical elastography at 39 weeks of gestation and the incidence of spontaneous labor within 7 days after the exam. (**A**) Bishop score 0–2, (**B**) Bishop score 3–5, (**C**) Bishop score 6–8. Palatal axis; cumulative spontaneous labor onset rate. Horizontal axis: days since the inspection.

We analyzed the association between cervical elastography findings at 39 weeks and the rate of onset of labor within 1 week, stratified by the Bishop score. At Bishop scores of 3–5 and 6–8, soft tissue elastography displayed a significantly increased rate within 7 days (p = 0.0007 and p = 0.03, respectively, Fig. 5).

The rate of the occurrence of spontaneous labor within 7 days from the 39-week pregnancy test was high in both VOLUSONS6 and ARIETTA60 compared to the soft tissue group and the hard tissue + medium-hard tissue group (p = 0.03 and p = 0.07, respectively; Supplementary Figure S3).

We performed a multivariate analysis using the Cox proportional hazard model to assess age, height, weight, BMI at the time of the examination at 39 gestational weeks, primipara vs. multipara, Bishop score (0–2 vs. 3–5 vs. 6–8), and elastography (hard tissue vs. medium-hard tissue vs. soft tissue) (Table 2). We found that the Bishop score (HR 1.81, 95% CI 1.40–2.34, p < 0.0001) and elastography (HR 1.86, 95% CI 1.46–2.35, p < 0.0001) were independently related to labor onset rate within 1 week after the examination.

**Table 2:** Association between findings at 39 weeks gestation and labor onset within 1 week; analysis by the Cox proportional hazard model.

	Univariate			Multivariate		
	HR	95% CI	p value	HR	95% CI	p value
Age	1.00	1.00–1.02	0.75	1.00	0.97–1.03	0.86
Height	1.02	0.99–1.05	0.26	0.98	0.83–1.16	0.83
Body weight	1.00	099–1.02	0.50	1.04	0.86–1.26	0.68
Body mass index	1.01	0.98–1.04	0.61	0.91	0.55–1.51	0.72
Obstetric history (primipara vs. multipara)	1.58	1.11–2.26	0.01	1.11	0.77–1.61	0.58
Bishop score (0–2 vs. 3–5 vs. 6–8)	2.92	1.40–3.43	< 0.0001	1.81	1.40–2.34	< 0.0001
Elastography (hard tissue vs. medium hard tissue vs. soft tissue)	2.04	1.64–2.54	< 0.0001	1.86	1.46–2.35	< 0.0001

*HR* hazard ratio, *CI* confidence interval.

Even in cases where cervical ripening was considered favorable based on elastography and the Bishop score, there were cases in which a cesarean section was performed due to fetal dysfunction or cephalopelvic disproportion. Therefore, no significant correlation was observed between elastography and the vaginal delivery rate (data not shown).

## Discussion

Although shear wave elastography using acoustic radiation force impulse (ARFI) has recently been developed as a method of elastography, the use of ARFI on the gestational uterus is not acceptable in Japan due to concerns about the safety of the fetus^9,10^. Therefore, strain elastography was used in this study. A qualitative assessment of cervical elastography by color has been reported^11–13^, but an attempt has recently been reported to quantitatively assess one area of the cervical canal as a strain ratio by comparing it with other areas, in order to determine its association with pregnancy outcome^14^. At the start of the present study, an attempt was also made to assess quantitative elastography. However, the results were not reproducible between different examiners (data not shown). To date, most cervical elastography studies have been reported as data from one or two examiners^14^, and validation of the interobserver variation was considered inadequate. Therefore, in the present study, we assessed the endometrium by color, similar to the method reported by Wozniak et al.^13^. This simple method had very little interobserver variation, and all six of the testers had consistent results within the first five rounds. A study^15^ involving seven centers and 602 patients to evaluate thyroid nodules by strain elastography also conducted a qualitative evaluation by color, and at the moment, qualitative evaluation by color might be the method that is less prone to interobserver variation problems and is easier to apply clinically.

Some articles have highlighted the usefulness of cervical elastography for the prediction of the preterm birth rate^13,16,17^, and others have discussed the relationship between cervical elastography before labor induction and the success rate of transvaginal delivery^18,19^. However, some authors have questioned the usefulness of cervical elastography during pregnancy^14^. We believe that preterm birth rates and success rates of vaginal delivery are easy-to-understand indicators of clinical benefit, but they are affected by factors other than cervical ripening, such as treatment, bony birth canal, and fetal status. In addition, those studies include bias because physicians know the results of cervical elastography and then perform the preterm birth treatment or made the delivery-style decision. Thus, whether cervical elastography can be a truly reliable test to determine the degree of cervical ripening has not yet been fully evaluated. One of the reasons why the value of cervical elastography is questioned is that the cervix is rarely biopsied during pregnancy, making it impossible to directly examine whether cervical elastography is correlated with histological cervical ripening findings. Therefore, in this study, we decided to compare cervical elastography with the Bishop score at term and the time to spontaneous labor.

Dilatation, station, and position in pregnancy pelvic examinations have been reported to be correlated with dilatation, fetal head-perineum distance, and posterior cervical angle by transvaginal ultrasonography, respectively^20^, but no direct comparison of cervical elastography and pelvic findings during pregnancy has been reported. We found that cervical elastography during pregnancy was strongly correlated with cervical stiffness at pelvic examination (Table 1). This result was not affected by the difference in the ultrasonic model, and our results show the reliability of cervical elastography. Interestingly, cervical elastography was strongly correlated with effacement and dilation but weakly correlated with station and position. This indicates that cervical elastography is more strongly correlated with factors that indicate cervical ripening than factors that indicate fetal position.

In this study, we have shown that cervical elastography can predict the time of onset of spontaneous labor, and cervical elastography at 39 weeks of pregnancy is correlated with the incidence of spontaneous labor within 7 days after the test, regardless of whether the woman is primipara or multipara for the first time (Figs. 3 and 4). We also found that adding cervical elastography to the Bishop score at 39 weeks gestation can be useful for predicting the incidence of spontaneous labor (Fig. 5). This result is thought to occur because only information on the surface can be obtained through palpation, while information on the hardness inside the cervical canal is added by performing elastography. Panelli et al. reported that women with > 1-cm dilation were more likely to experience spontaneous labor than those with < 1-cm dilation at 39 weeks^21^. Rozenberg et al. reported that the Bishop score, ultrasonographic measurement of cervical length, and fetal fibronectin are valuable for predicting the onset of spontaneous labor within 7 days between 39 weeks 4 days gestation and 40 weeks 3 days gestation^22^. These reports support our findings that predicting the onset of spontaneous labor from cervical ripening was possible only at 39 weeks. Limitations include: (1) a relatively small sample size; (2) the instrument changed midway through although the same trend was obtained; and (iii) the elastography was evaluated qualitatively and could not be analyzed as precisely as the cut-off setting by strain ratio.

In conclusion, we directly compared cervical elastography with the Bishop score and evaluated its significance using the bias-free index of the time to the onset of spontaneous labor at term. We have shown that cervical elastography at 39 weeks of gestation is useful for predicting the onset of spontaneous labor. Predicting the time of the onset of spontaneous labor is helpful in preparing for and planning scheduled deliveries. A limitation of this study is that the sample size is relatively small. Thus, to incorporate the results of this study as a standard clinical method in the future, a prospective study with a greater sample size is now required.

## Materials and methods

### Patients

The subjects included 147 primiparas and 91 multiparas who delivered at our hospital during the 2 years from January 2017 to December 2018. Sixteen additional cases were added in July 2020 to assess the reproducibility of elastography. Informed consent was obtained from all subjects with the approval of our ethics committee. This study was approved by the Institutional Review Board of Kindai University Faculty of Medicine (28–180). All research was performed in accordance with the Ethical Guidelines for Medical and Health Research Involving Human Subjects.

A pelvic examination was conducted at weekly pregnancy checkups from 36 to 40 weeks of gestation, and elastography was used to evaluate cervical stiffness by transvaginal ultrasonography. From 2017 to 2018, a total of 769 examinations were performed by Y.Y., M.S., R.S., K.Y., R.F., and A.S. Before starting this study, Y.Y. and M.S. performed elastography examinations together using a transvaginal probe in ten pregnant women to ensure that the results in the same case were stable and consistent with each other. Subsequently, when R.S., K.Y., R.F., and A.S. participated in the study, they (also together with Y.Y. or M.S.) performed elastography examinations using a transvaginal probe on five or more pregnant women to ensure that the results in the same case were consistently obtained and that the results were consistent across examiners. It was also confirmed that there was no difference in the results between examiners when the ultrasonic equipment was changed. In addition, in July 2020, we examined the concordance rate of elastography assessments between Y.Y. and M.S., R.S., or K.Y. in 16 cases. The number of observed agreements was 88%, and the weighted Kappa value was 0.862 (Supplementary Table S1)^23^.

The inclusion criteria were cases in which the management of pregnancy and delivery was performed at our department after 36 weeks. Exclusion criteria included patients diagnosed with threatened preterm labor by 36 weeks and treated with ritodrine hydrochloride or magnesium sulfate, and/or patients with multiple pregnancies.

### Elastography and scoring of pelvic examination findings

Until May of 2018, VOLUSONS6 (GE Healthcare, Japan, Tokyo) was used, and beginning in June of 2018, ARIETTA60 (Hitachi, Tokyo) was used to replace the ultrasonic equipment at the facility. Both are strain elastography devices.

Elastography was qualitatively assessed by color. First, a transvaginal probe was placed on the anterior surface of the cervix as a lithotripsy with the bladder emptied, and a sagittal section of the cervix was obtained. The mucosa in the cervix was drawn using an echo along the length of the cervix. The probe was moved approximately one centimeter four to five times, and the cycle of compression and decompression was repeated. The region of interest was determined by the dominant color of the cervical gland as a whole and at 1 cm in diameter on the side of the inner uterus, according to the manufacturer’s protocol, with a pressure indicator bar (VOLUSONS6) and a waveform display (ARIETTA60).

In VOLUSONS6, green indicates hard tissue, yellow indicates medium-hard tissue, and red indicates soft tissue (Fig. 1). In ARIETTA60, blue indicates hard tissue, green indicates medium-hard tissue, and red indicates soft tissue. Elastography was assessed using the qualitative results.

To perform Spearman rank correlation analysis, the numbers zero for hard tissue, 1 for medium-hard tissue, and 2 for soft tissue were used.

Stiffness was not measured numerically among the findings of the pelvic examination; thus, it was quantified as follows: zero for hardness, 1 for moderate, and 2 for soft. Furthermore, the position of the cervix was quantified numerically: zero for posterior, 1 for the middle, and 2 for anterior. The effacement (%), dilation (cm), station (cm), and Bishop score^1^ of the pelvic examination findings were used as their actual values. The correlation between elastography and the pelvic examination findings from the same examination were analyzed using a Spearman’s correlation (Table 1).

### Comparison of the number of days from the examination date to the onset of natural labor

In Japan, pregnancy screening is performed weekly after 36 weeks of pregnancy^24^. In this study, we analyzed the incidence of spontaneous labor within 7 days after the examination, starting at the consultation between 36 and 40 weeks. Among the cases without spontaneous labor, the cases in which labor was induced were treated as censored (i.e., no event) at the beginning of labor induction. Similarly, the cases in which a cesarean section was performed (without spontaneous labor) were treated as censored at the start of the cesarean section.

### Statistical analysis

Spearman’s correlation coefficient was used for the correlation analysis between the elastography and pelvic findings, which included stiffness, hardness, effacement, dilatation, station, and the Bishop score. The Kaplan–Meier curve approach was used to estimate the rate of cumulative spontaneous labor (Figs. 2, 3, 4 and 5), and the log-rank test was performed to compare the groups stratified by elastography findings (hard tissue, medium-hard tissue, or soft tissue) and the Bishop score (0–2, 3–5, or 6–8).

Also, to identify the factors involved in the time to labor onset, variates such as maternal age, height, weight (at the time of examination), and BMI were treated as continuous variables, obstetrical history (primipara vs. multipara) was treated as a dichotomous variable, and the Bishop scores (0–2 vs. 3–5 vs. 6–8) were treated as scale variables, which were generally known as maternal factors. Clinical findings associated with the time to labor onset were applied to the univariate Cox proportional hazards model along with the color from the elastography. Then, we calculated the hazard ratios (HRs), which indicated the ratio of labor onset rate per unit of time or per change or per unit of variable or across groups. To consider the confounding factors, the covariates described above were applied to the multivariate Cox proportional hazards model to examine the association with the color from the elastography in regard to the time to labor onset (after these covariates were adjusted).

The analysis using the Cox proportional hazard model was performed using R version 3.5.3 (https://www.R-project.org/). Other statistical analyses were performed using Prism Version 6.0 (GraphPad Software, San Diego). A p-value of < 0.05 was considered statistically significant.

## Supplementary information

Supplementary Figure S1.

Supplementary Figure S2.

Supplementary Figure S3.

Supplementary Table 1.

Supplementary Information.
