# Supplementary figures and images for: Relationship between cervical elastography and spontaneous onset of labor
Source: Sci Rep. 2020 Nov 12;10:19685. doi: 10.1038/s41598-020-76753-4 (PMC7661529; doi:10.1038/s41598-020-76753-4)

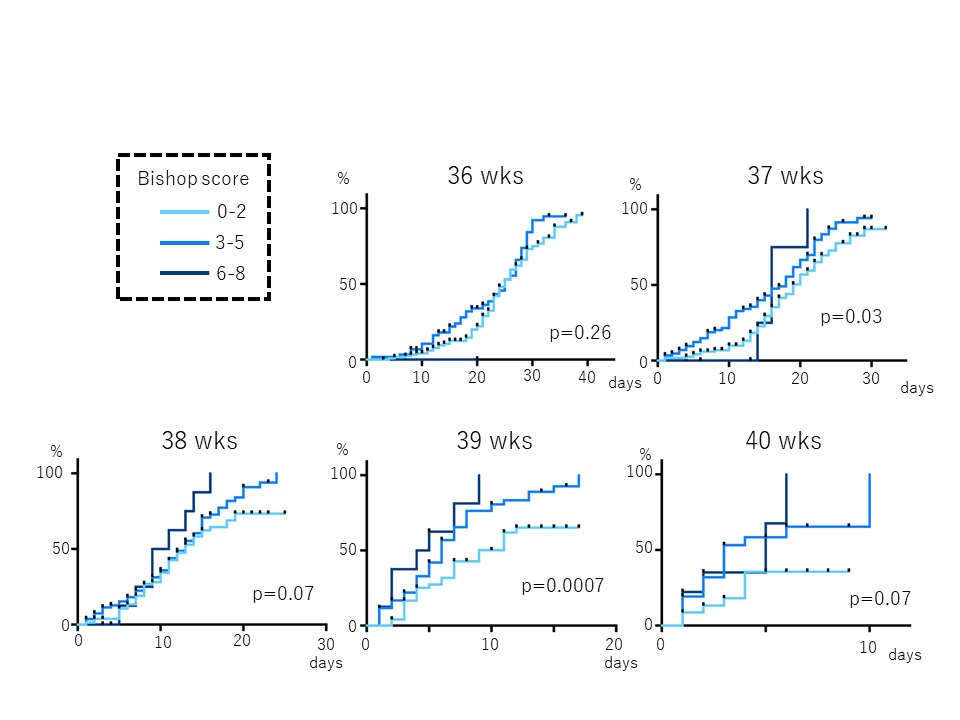

Supplement: Supplementary file 1 — Supplementary Figure S1. [file 41598_2020_76753_MOESM1_ESM.tif]

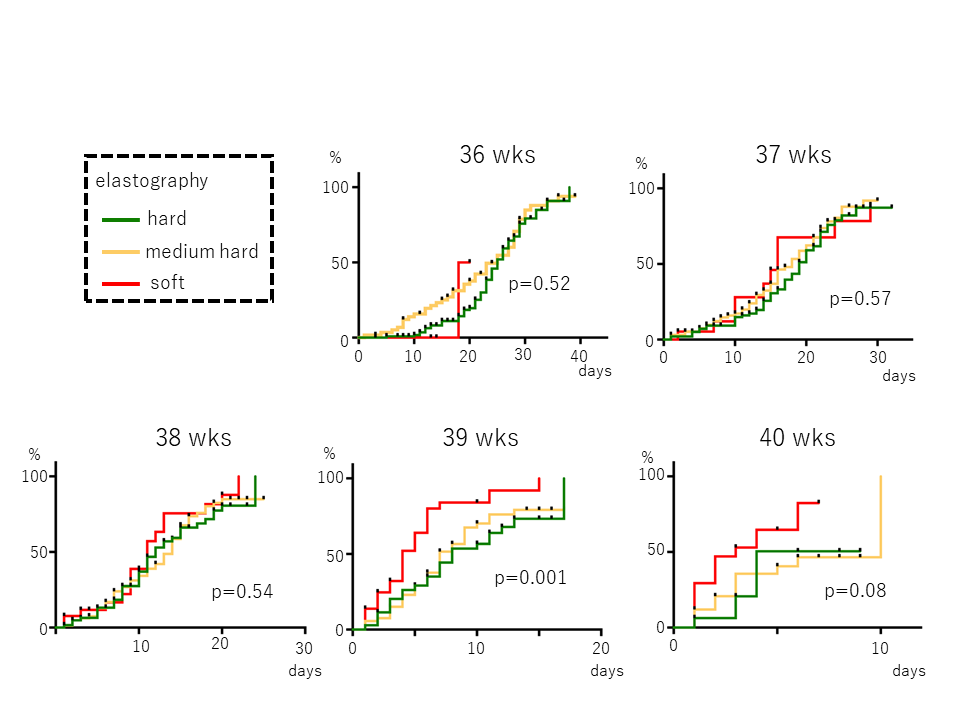

Supplement: Supplementary file 2 — Supplementary Figure S2. [file 41598_2020_76753_MOESM2_ESM.tif]

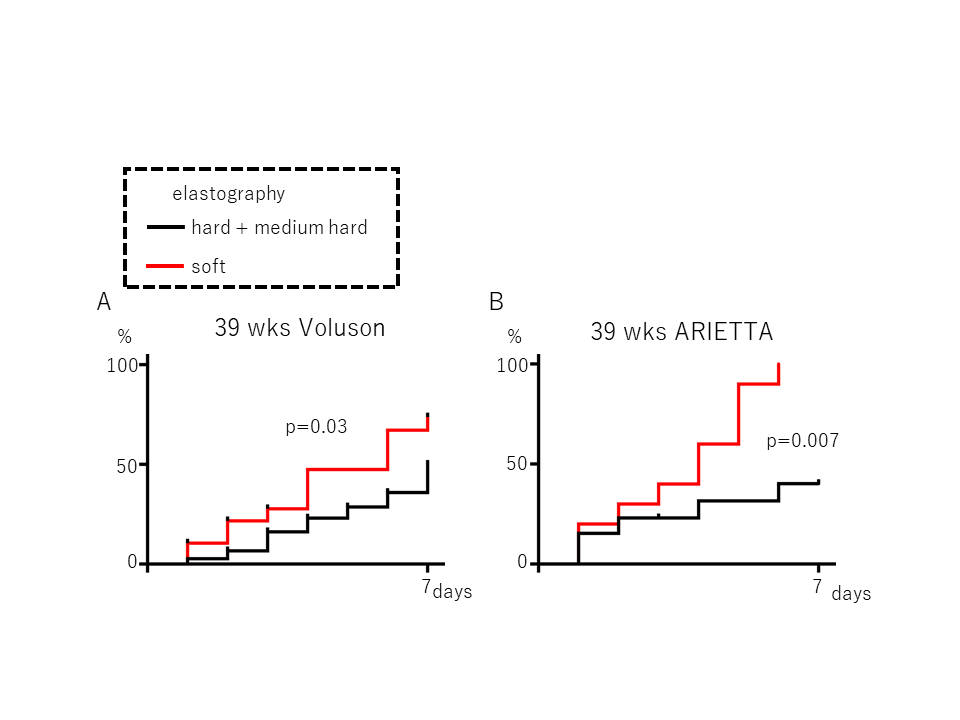

Supplement: Supplementary file 3 — Supplementary Figure S3. [file 41598_2020_76753_MOESM3_ESM.tif]
