# Supplementary material for: Relationship between cervical elastography and spontaneous onset of labor
Source: Sci Rep. 2020 Nov 12;10:19685. doi: 10.1038/s41598-020-76753-4 (PMC7661529; doi:10.1038/s41598-020-76753-4)
Supplement: Supplementary file 4 — Supplementary Table 1. [file 41598_2020_76753_MOESM4_ESM.docx]

|  |  | R.S. or M.S., or K. Y. | | |
| --- | --- | --- | --- | --- |
|  |  | Soft | moderate | hard |
| Y.Y. | Soft | 5 | 0 | 0 |
|  | moderate | 0 | 4 | 0 |
|  | hard | 0 | 2 | 5 |

Supplementary Table 1. Reproducibility of elastography.

We looked at the concordance rate of the tests in 16 pregnant women between 36 and 39 weeks in July 2020. First, Y.Y. performed the test, and with the result hidden, then M.S., R.S., or K.Y. performed the test on the same pregnant woman. As a result, the ratio of the observed agreements was 88% (14/16), and the weighted kappa statistic was 0.862, which was a high concordance rate. In particular, the judgment of soft (red) was in 100% agreement.
