# Supplementary material for: Relationship between cervical elastography and spontaneous onset of labor
Source: Sci Rep. 2020 Nov 12;10:19685. doi: 10.1038/s41598-020-76753-4 (PMC7661529; doi:10.1038/s41598-020-76753-4)
Supplement: Supplementary file 5 — Supplementary Information. [file 41598_2020_76753_MOESM5_ESM.docx]

Supplementary Figure 1. Relationship between the Bishop score and the number of days until the onset of spontaneous labor. Palatal axis; cumulative spontaneous labor onset rate. Horizontal axis: days since the inspection.

Supplementary Figure 2. Association of cervical elastography with the number of days from examination to the onset of spontaneous labor. Palatal axis; cumulative spontaneous labor onset rate. Horizontal axis: days since the inspection.

Supplementary Figure 3. Association between cervical elastography at 39 weeks of gestation and the incidence of spontaneous labor within 7 days after the exam. We examined whether differences in ultrasonic models affected the results. Elastography results were combined for hard tissue and medium-hard tissue cases and compared with soft tissue cases. A) VOLUSONS6. B) ARIETTA60. Palatal axis; cumulative spontaneous labor onset rate. Horizontal axis: days since the inspection
